# Synthesis, characterization and application of new adsorbent composites based on sol-gel/chitosan for the removal of soluble substance in water

**DOI:** 10.1016/j.heliyon.2022.e09444

**Published:** 2022-05-17

**Authors:** Jonatan Rafael de Mello, Thaís Strieder Machado, Larissa Crestani, Ingridy Alessandretti, Giovana Marchezi, Flávia Melara, Marcelo Luis Mignoni, Jeferson Steffanello Piccin

**Affiliations:** a Postgraduate in Food Science and Technology, Faculty of Agronomy and Veterinary Medicine, University of Passo Fundo, BR 285, km 171, Passo Fundo, RS, Brazil; b Postgraduate in Civil and Environmental Engineering, Faculty of Engineering and Architecture, University of Passo Fundo, BR 285, km 171, Passo Fundo, RS, Brazil; c Chemical Engineering Course, Faculty of Engineering and Architecture, University of Passo Fundo, BR 285, km 171, Passo Fundo, RS, Brazil; d Department of Food and Chemical Engineering, University of Regional Integrated of Alto Uruguay and the Missions, Avenue Sete de Setembro, 1621, Erechim, RS, Brazil

**Keywords:** Xerogel, TEOS, Amine groupings, Tartrazine yellow, Adsorption

## Abstract

In this work, new adsorbent composites from the silica precursor tetraethyl orthosilicate (TEOS) and chitosan have been successfully synthesized, denominated 20%Chi, 30%Chi and 40%Chi. The composites presented enhanced chemical and physical characteristics, with emphasis on the high surface areas between 374.94 m^2^/g to 886.31 m^2^/g. The application of the composites in the model system (TY - Tartrazine yellow dye), presented adsorption capacities dependent on the amount of chitosan in the composite (40%Chi > 30%Chi > 20%Chi). However, from the experimental data of the constituent materials, 30%Chi provided the greatest increase in the adsorption capacity in the monolayer, with values of 36%. This demonstrates that the amount of chitosan in the compound alters the arrangement of adsorption sites. The 30%Chi composite presented life cycle superior to 10 reuse cycles.

## Highlights

• The addition of silica provided better physical and chemical properties to the developed composites.
• The synthesis of the 30%Chi composite enabled an increase in the adsorption capacity of the TY dye.
• The 30%Chi composite obtained a surface area of 886.31 m^2^/g.
• The 30%Chi composite was useful for more than 10 adsorption and desorption cycles.

## Graphical abstract

**Figure:**
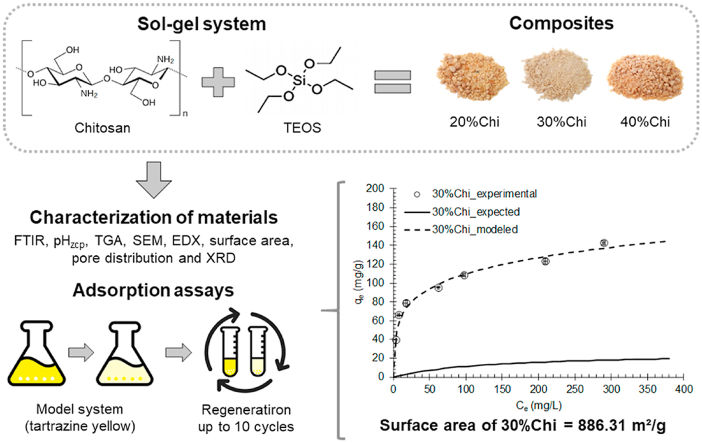

## 1 Introduction

Dyes, agrochemicals, personal care products, drugs, among other substances are a class of contaminants that have been attracting the attention of the scientific community [1, 2, 3, 4, 5, 6, 7, 8, 9, 10, 11]. These substances, when present in the environment in an uncontrolled way, are called emerging contaminants, which conventional effluent treatments are not able to efficiently remove.

Adsorption has been evaluated as a promising technique for the removal of emerging contaminants. This is due to the fact that adsorption is efficient for the removal of these contaminants even at low concentrations, in the order of μg/L and ng/L, situations in which conventional processes have less removal efficiency [11]. In addition, the adsorption equipments are simple and relatively easy to operate. Another advantage is the fact that they require little energy, which makes the operating cost associated with the material used as an adsorbent. In the latter case, the regeneration and reuse of the adsorbent material are issues that must be considered in its choice.

Activated carbon is the most used commercially adsorbent material. However, due to the new challenges for the effective removal of emerging contaminants, different materials have been used in natural or synthesized form for this purpose. In this case, materials of natural origin [12], industrial waste [10, 13, 14, 15] and composite materials can be mentioned [1, 2, 3, 7, 16, 17, 18]. Dyes are seen as a good model system for the evaluation of new adsorbent materials. This is because they behave similarly in water and have chemical groups characteristic of emerging contaminants, such as aromatic rings, aromatic amines, azo bonds, sulfonated groups, among others. In addition, azo dyes (-N=N-) are known to cause several problems to human health and to the ecosystem, such as the case of the food dye TY [19, 20, 21].

Chitosan, a fishing residue obtained from shrimp shells, stands out among alternative materials used as unconventional adsorbents. Chitosan is mainly used to remove contaminants from industrial effluents, such as textile and alimentary dyes and toxic metals [2, 3, 7, 22, 23, 24, 25, 26]. Its main characteristic is to have amine groups, which act as adsorption sites with polycationic nature, such as polyelectrolyte and chelating agent. However, chitosan in its natural form is soluble in an acidic medium, has a low surface area and porosity, which limits its application as an adsorbent.

The synthesis of adsorbent composites can be performed by the sol-gel system. Such a system allows the production of materials with various structural compositions, with high degree of purity and high homogeneity from salt and alkoxide precursors [27, 28, 29]. It is commonly used as a method to obtain glassy and ceramic materials through a “sol” solution, followed by its gelation and removal of the liquid phase. The materials obtained can be denominated alcohologel, sonogel, xerogel, airgel or cryogel [30]. In general, the sol-gel system allows obtaining particulate materials, with high surface area and high mechanical properties [31], characteristics desired for an adsorbent. Thus, the production of synthesized composites from chitosan and silica, precursor to alkoxides, can minimize the disadvantageous characteristics of chitosan in front of its application as an adsorbent.

Therefore, the objective of this study was to develop adsorbent composites based on silica and chitosan using the sol-gel system, verifying its potential for removing emerging contaminants in water. For this, chitosan-based composites were produced with different silica content. The materials were characterized and submitted to adsorption and reuse experiments in aqueous solutions.

## 2 Materials and methods

### 2.1 Chitosan production

Chitosan was obtained from shrimp shells by the procedure described by Weska et al. [32], according to adaptations suggested by Moura et al. [33]. This process consists in the steps of demineralization, deproteinization, deodorization, drying of chitin, deacetylation of chitin and purification of chitosan.

The demineralization was performed at room temperature and agitation of 60 rpm for 2 h using 2.5% HCl (v/v) in the proportion of 2 L/kg of shrimp shells. After, the shells were deproteinized in a 5% NaOH solution (w/v) in the proportion of 3 L/kg of shrimp shell, with agitation of 60 rpm for 2 h. Deodorization was performed with a 0.36% (v/v) NaClO solution in the proportion of 5 L/kg of shrimp shell, keeping the material under agitation of 60 rpm for 3 h. After each step, the material was washed with tap water to remove the excess reagent. The final material originates the chitin, which was dried by convection at 60 °C for 24 h (Tecnal, TE-394) and crushed.

The chitin thermochemical deacetylation was carried out in a reactor with open reflux (own confection in stainless, diameter of 22 cm and 6 L of useful capacity) coupled to a system of mechanical agitation and a system of total condensation of vapors generated. In this step, a 45% NaOH solution (w/v) in the proportion of 20 mL/g of chitin was used. The system was heated to the boiling temperature of the solution (approximately 115 °C) for 4 h. After cooling, the NaOH solution was poured and the material was washed with tap water until the pH was close to neutral (7.5–8.0), resulting in unpurified chitosan.

Chitosan was purified by solubilization in a 2% (v/v) acetic acid solution, with 100 mL of solution for each 1 g of solids, and kept under agitation at 60 rpm for approximately 12 h. This solution was centrifuged at 3500 rpm for 20 min (Eppendorf, 5810r) and the solid material was discarded. The pH of the supernatant was adjusted to 12 with 2 mol/L NaOH (Digimed, DM-22), for the chitosan precipitation. After complete precipitation (1–2 h), the pH of the suspension was adjusted to 7.5 with 1 mol/L HCl and then centrifuged at 3500 rpm for 20 min (Eppendorf, 5810r), obtaining the purified wet chitosan. The chitosan obtained was lyophilized (Terroni, LS 3000) and ground in a mortar. The biopolymer samples were stored in vacuum packaging and kept at room temperature until later use. Chitosan was characterized by potentiometric titration [34, 35] with a degree of deacetylation of 91.22%.

### 2.2 Synthesis of the adsorbent composites by the sol-gel system

The adsorbent composites of this study were developed according to the patent of invention n° BR1020210030291 [36]. Briefly, the silica precursor (tetraethyl orthosilicate or TEOS, Sigma Aldrich, analytic degree) was mixed with a hydrolysis solution composed of ethanol, water and hydrochloric acid, in the molar proportion of 1:4.55:2.46:0.022, respectively, and subjected to pre-hydrolysis at 35 °C at 150 rpm for 2 h (Tecnal, TE-421). Concurrently, a solution containing 2% chitosan (w/v) in 2% acetic acid (w/v) was prepared. After TEOS hydrolysis and chitosan solubilization, the solutions were mixed and stirred for approximately 1 h, and atomized in the form of a spray on the polycondensation solution (0.5 mol/L ammonium hydroxide). Atomization was performed with a nozzle of the type external mixing double-fluid, with a bore diameter of 1.2 mm, atomization airflow of approximately 40 L/min and solution flow of 0.5 L/h. The amounts of TEOS, chitosan and ammonium hydroxide are described in Table 1. The volume of the polycondensation solution (Table 1) was determined from a stoichiometric balance to neutralize the volume of acetic acid used in the solubilization of chitosan and to maintain the same amount of excess NH_4_OH in all formulations, considering the methodology of Castro [37].

**Table 1:** Stoichiometric and real mass of each composite synthesized in relation to the amount of 1 g chitosan.

	Composite		
	20%Chi	30%Chi	40%Chi
TEOS volume (mL) in the formulation	15	10	5
Ammonium hydroxide volume (mL) 0.5 mol/L in the formulation	240	185	150
Composite mass expected by stoichiometry (g)	5.03	3.69	2.34
Chitosan percentage expected by stoichiometry (%, w/w)	19.9	27.1	42.7
Silicate percentage expected by stoichiometry (%, w/w)	80.1	72.9	57.3
Composite mass obtained (g)	7.30	5.39	2.58
Chitosan percentage determined by material balance (%, w/w)	13.7	18.6	38.8
Silicate percentage determined by material balance (%, w/w)	86.3	81.4	61.2

The resulting mixture went through a thermal aging step in an oven at 35 °C until complete drying (DeLeo, DL-SED), for approximately 5 days. After the first 24 h, the excess of water was removed, due to the condensation reaction generating water as a by-product. With the complete reaction and drying, the material was washed in a Soxhlet extractor with acetone P.A. for 2 h, rinsed with distilled water to remove possible washing residues and dried in a humidity-free atmosphere under vacuum for 2 days.

The materials were named 20%Chi, 30%Chi and 40%Chi, which represent approximately the amount of chitosan expected in the material, considering the complete hydrolysis and polycondensation of the silica precursor used (stoichiometric balance). In addition, a material without the addition of chitosan (0%Chi) was developed for experimental control, according to the methodology described in Castro [37]. The composition of the materials obtained at the end of the process was determined by weighing, relating the mass of chitosan used in each formulation, thus, the percentage of chitosan in each of the compositions was determined.

### 2.3 Characterization of the adsorbent composites

FTIR analysis was performed using the Attenuated Total Reflectance technique (ATR, Agilent Technologies, Cary 630) in the range of 4000 to 650 cm^−1^, scanned 48 times each spectrum with a resolution of 4 cm^−1^. The processing of the spectrum was performed with MicroLab software version B.5 (Agilent Technologies).

The identification of the material pH_zcp_ was carried out according to Newcombe et al. [38], with adaptations. Initially, 0.05 g of the material was added in 20 mL of 0.05 mol/L NaCl solution under nine different initial pH conditions (Digimed, DM-22), in the ranges of 2.0–10.0, adjusted with 0.1 mol/L HCl and 0.1 mol/L NaOH solutions. The mixture was stirred for 24 h at 100 rpm and at 25 °C (Tecnal, TE-421) and, subsequently, the final pH was measured.

Thermogravimetric analyses (TGA) were performed in a nitrogen inert atmosphere at a 20 mL/min flow rate in the range of 150 °C–824 °C, using a heating rate of 10 °C/min (Netzsch, STA 449 F3).

Scanning electron microscopy (SEM) analyses were performed with voltage acceleration from 10 kV to 15 kV and magnification range of 40–5000 times (Tescan, VEJA 3). The samples were metallized by evaporating gold at high vacuum (Quorum, Q150R ES). In parallel, energy dispersive X-ray spectroscopy analysis (EDX, Oxford Instruments, X-Max 20) coupled to SEM was performed.

The surface area and the pore distribution of the materials were determined from nitrogen adsorption isotherms at -196.15 °C, with a relative pressure varying from 0 to 0.99 (Quantachrome, Nova 2200e). The sample was prepared at a temperature of 50 °C under vacuum for 12 h, to remove the adsorbed molecules on the surface without compromising the organic structure of the composite. The specific surface area and pore distribution were determined using the BET (Brunauer-Emmett-Teller) and BJH (Barrett-Joyner-Halenda) theories, respectively.

The crystal structures of the samples were investigated by X-ray diffraction (XRD), with Cu-Kα radiation (1.54184 Å) in a 30 kV and 10 mA configuration (Bruker, D2 PHASER).

### 2.4 Adsorption experiments

Adsorption experiments were conducted using a system of aqueous solutions of the TY dye (CAS n° 1934-21-0, 534.35 g/mol, food-grade and 90% purity) as a contaminant model. A concentrated solution of 1 g/L was prepared. After, the dilutions were made to obtain the desired concentrations, using distilled water. The concentration of the dye in aqueous solution was determined by spectrophotometry at 429 nm (Tecnal, UV-5100), according to the Lambert-Beer law (Absorbance = 0.0352∗[TY], R^2^ > 0.999). All adsorption experiments were performed in duplicate and represented by the mean and standard deviation.

Initially, the ideal adsorption pH was evaluated in the range of 2.5–8.5. For this, 0.05 g of adsorbent was mixed with 5 mL of McIlvaine buffer solution (at the studied pH), and left in contact for a few minutes for the pH correction and temperature setting. Subsequently, 50 mL of the solution containing 100 mg/L of the dye was added. The experiments were carried out on an orbital shaker table (Tecnal, TE-421), at 100 rpm and at 25 °C. The experiment was conducted for 2 h. Afterward, the aliquots were removed and centrifuged (BioPet, 8011154V), to determine the residual dye concentration.

Adsorption isotherms were performed similarly. However, in this case, the initial concentration of the dye was varied in a range between 50 mg/L to 400 mg/L, obtaining different equilibrium conditions. For each experiment, 0.05 g of the adsorbent, 5 mL of the McIlvaine buffer solution and 50 mL of the dye solution were added. The system was stirred at 100 rpm in an orbital shaker table (Tecnal, TE-421) until the solution reached the equilibrium concentration (approximately 24 h). The adsorption isotherms were carried out at 15, 25 and 35 °C.

The adsorption isotherms were analyzed using isotherm models by Langmuir [39], Freundlich [40] and Redlich & Peterson [41], according to the equations described in the supplementary material (Equations a.1, a.2 and a.3). The adsorption isotherm models were calculated by non-linear regression using the algorithm “lsqnonlin” of MATLAB® Software (Free Trial Version, MathWorks®, United States) through of the minimization of the residual sum of the squares [42, 43]. The regression analysis was performed using the statistical parameters of the coefficient of determination (R^2^) and adjusted coefficient of determination (adjusted R^2^).

The analysis of the adsorption thermodynamics was performed through the calculation of values of the Gibbs free energy (ΔG^0^), the adsorption enthalpy (ΔH^0^) and the adsorption entropy (ΔS^0^).

Similarly, the adsorption kinetics was performed using 250 mL of an aqueous solution containing 100 mg/L of TY dye, 0.3 g of the adsorbent and 25 mL of McIlvaine buffer solution (at the studied pH). The assays were performed at 100 rpm and at 25 °C (Tecnal, TE-421). The supernatant concentration was evaluated for a period of up to 360 min. The adsorption kinetics were analyzed using kinetic models by Pseudo-first-order [44] and Pseudo-second-order [45, 46], according to the equations described in the supplementary material (Equations a.4 and a.5).

#### 2.4.1 Adsorption capacity expected by the adsorbent composite

To verify if the synthesis process contributes in the adsorption capacity increment of the materials, adsorption isotherms of chitosan in natural form and of the 0%Chi material were performed. The isotherms were evaluated by the proposed models and the expected adsorption capacity (q_expected_) for each of the composites was predicted according to Eq. (1).

(1) $$q_{expected}=\left(P_{q}\times q_{q}\right)+\left(P_{x}\times q_{x}\right)$$

where, P_q_, P_x_ is the proportion (%, w/w) of chitosan and silica, respectively; and q_q_, q_x_ is the adsorption capacity (mg/g) of chitosan and silica, respectively, calculated from the best fit adsorption isotherm model. The mass of chitosan and silica were estimated by mass balance of the components in the final product.

### 2.5 Life cycle of the adsorbent composite

The life cycle of the absorbent was evaluated through cyclic adsorption and desorption experiments. In Falcon tubes with a useful volume of 50 mL, were added 0.02 g of adsorbent and 3 mL of buffer solution at the ideal pH of the adsorption experiments. Then, was added 30 mL of the contaminant solution with a concentration of 200 mg/L. The tube was fixed and kept under agitation at 100 rpm, at 25 °C for 3 h (Tecnal, TE-421). Afterward, the contaminant solution was removed by centrifugation at 3000 rpm for 10 min (Quimis, Q222T204) and an aliquot was removed to determine the concentration of the remaining contaminant.

For desorption, 5 mL of 0.05 mol/L NaOH solution was added to the solid material and kept under stirring at 100 rpm, at 25 °C for 10 min. The NaOH solution was removed by centrifugation, then the material was washed twice with 10 mL of demineralized water. The contaminant concentration in the desorption and in the washing liquid was determined by spectrophotometry. The adsorption capacity per cycle was calculated by Eq. (2) and the regeneration by Eq. (3).

(2) $$Adsorption\;capacity\left(\frac{mg}{g}\right)=\left(\frac{C_{initial}-C_{final}}{m}\right)\times V$$

where, C_initial_ is the initial concentration of the contaminant (mg/L), C_final_ is the final concentration corresponding to each adsorption cycle (mg/L), m is the mass of adsorbent in dry base (g), and V is the volume of the liquid phase (L).

(3) $$Regeneration\left(\%\right)=\frac{m_{des}}{m_{ads}}\times 100$$

where, m_des_ is the desorbed mass in each desorption cycle (mg) and m_ads_ is the adsorbed mass in each adsorption cycle (mg).

## 3 Results and discussion

### 3.1 Synthesis and characterization of composites

The synthesis method of the present work aimed to increase the availability of amine groups from chitosan. The technique used was sol-gel, having TEOS as a silica precursor. According to this method, two steps occur simultaneously in the silica precursor: the hydrolysis of silanol (Eq. (4)) and the polycondensation reaction of silicates (Eq. (5)). In the first reaction, TEOS is hydrolyzed to orthosilicic acid (Si(OH)_4_) in aqueous medium. The addition of chitosan with TEOS during hydrolysis leads to the formation of an inorganic network with strong chemical bonds between silicate groups and weak phase interactions, such as hydrogen bonds, which leads to the nucleation of silica into macromolecules. The acid pH is also a factor that favors the hydrolysis of the precursor, because the chitosan amino group is protonated and forms positively charged NH_3_^+^, which favors hydrogen bonds [27, 47]. In sequence, the polycondensable orthosilicic acid forms a three-dimensional network of silicate ([SiO_2_]n). In this stage, a three-dimensional vacant silicate network is formed, which gives space for the formation of a small chitosan film. Thus, chitosan can physically adhere to the three-dimensional silicate structure. Furthermore, the amino groups of chitosan can assist in the hydrolysis of TEOS and in the condensation of the formed silanol groups, forming Si–O–C cross-links between silanol groups of silica with the carbonyl groups of the polymer [27].

(4) $$SiC_{8}H_{20}O_{4}+4H_{2}O\to Si\left(OH_{4}\right)+4C_{2}H_{5}OH$$

(5) $$nSi{\left(OH\right)}_{4}\to \left[SiO_{2}\right]n+\left(2n\right)H_{2}O$$

Considering the amounts of TEOS and chitosan used in each of the formulations and the reactions described in Eqs. (4) and (5), Table 1 shows the formulation of the composites developed with stoichiometric basis (% expected, w/w) and based on the mass of material obtained at the end of each formulation (% real, w/w).

Table 1 shows the stoichiometric and real mass for each composite. It can be observed that the mass of the material obtained is higher than expected by the reaction stoichiometry. Suggesting that the TEOS hydrolysis may have occurred incompletely, generating silanol residues in the inorganic silica network, which were not eliminated in the washing of the material. In addition, the incomplete polycondensation of silicic acid can lead to the formation of small size structures, for example, silicon oxide, which can be charged by the washing process.

**Figure 1:**
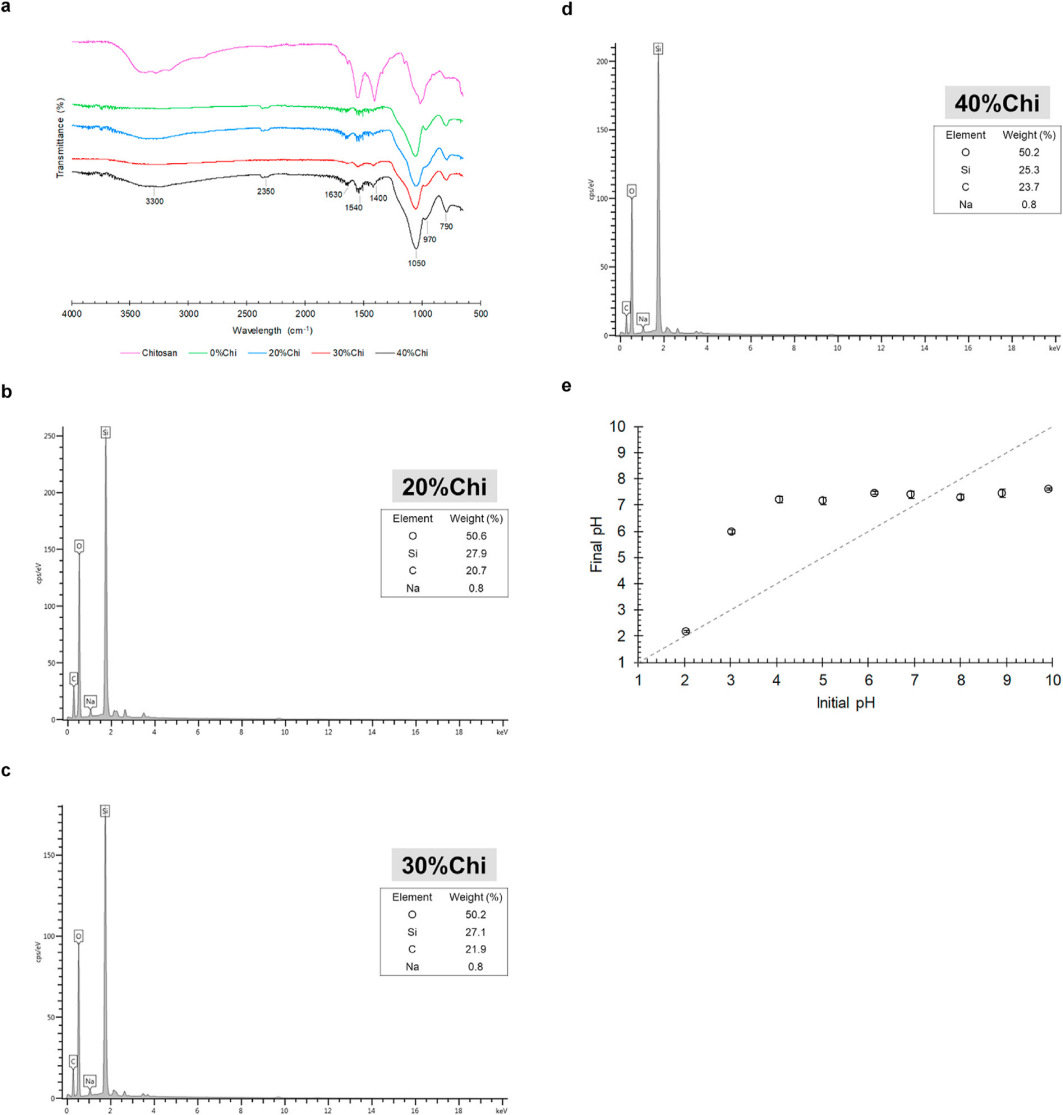
Chemical characterization of the developed composites, being: (a) FTIR spectrum, (b–d) EDX spectrum and (e) pH_zcp_ of the 30%Chi composite.

Figure 1 shows the chemical characterization (FTIR, EDX and pH_zcp_) of the synthesized materials. In the Figure 1 (a), the characteristic peaks of chitosan in natural form are observed in the bands of 3369 cm^−1^ (stretching of the O–H bond), 1017 cm^−1^ (stretching of the C–O–C bond), 2922 cm^−1^ (stretching of the N–H bond of primary amine), 1558 cm^−1^ and 1407 cm^−1^ (deformation of the amine) [27, 48, 49, 50].

In the spectrum of the composite without chitosan (0%Chi), the characteristic signs Si–OH of the silica (TEOS) can be observed in the bands of 2361 and 969 cm^−1^ respectively, which are stretching of the silanol groups present in the silica [51, 52, 53, 54]. The vibration from the stretching of the Si–O–Si group can be identified in the bands of 1056 and 1093 cm^−1^. The presence of these bands in the FTIR spectrum (Figure 1 (a)), confirms that the silica samples in the sol-gel system were obtained satisfactorily [51, 52, 53, 55].

All composites synthesized with the addition of chitosan, there is a spectral behavior similar to the control sample (0%Chi). Signs related to the deformations of primary amines (1558 and 1407 cm^−1^) are more pronounced in composites with higher amounts of chitosan. In addition, there is an overlapping of the C–O–C and C–H bond stretch in the 1017 and 797 cm^−1^ bands, by the group Si–O–Si of silica. The signs at approximately 970 cm^−1^, mainly for the 30%Chi and 40%Chi composites, suggest the interactions between the residual silanol groups and chitosan.

The EDX analysis (Figure 1 (b-d)) is in accordance with the data obtained from the FTIR spectrum. It is observed that the composites developed are essentially constituted by oxygen (O), silica (Si) and carbon (C). The high peak of Si in the EDX spectrum suggests that the composites consist predominantly of silica.

The influence of pH over the adsorbent composite occurs concerning its surface load. According to Yagub et al. [56], pH_zcp_ is a parameter that describes the variation of surface loads. According to Figure 1 (e), there is no change in the pH of the solution at pH 7.4, indicating that the surface loads of the composite are completely balanced. At lower values of pH compared to these, an increment in the final pH of the solutions is observed. Indicating that H^+^ ions leave the solution to protonate the surface of the adsorbent material. In these conditions, the composite has a greater affinity for anionic compounds. Otherwise, above pH_zcp_, OH^−^ ions react with the adsorbent surface. Leading to negative charges and more interaction with cationic compounds [57]. Similar results were found by Andrade Júnior [58], who synthesized an adsorbent material based on silica/epoxy/chitosan to remove Hg (II) in aqueous medium, having a pH_zcp_ of 7.62. This characteristic of the materials is due to the protonation of chitosan in acidic media and deprotonation in basic media [59].

**Figure 2:**
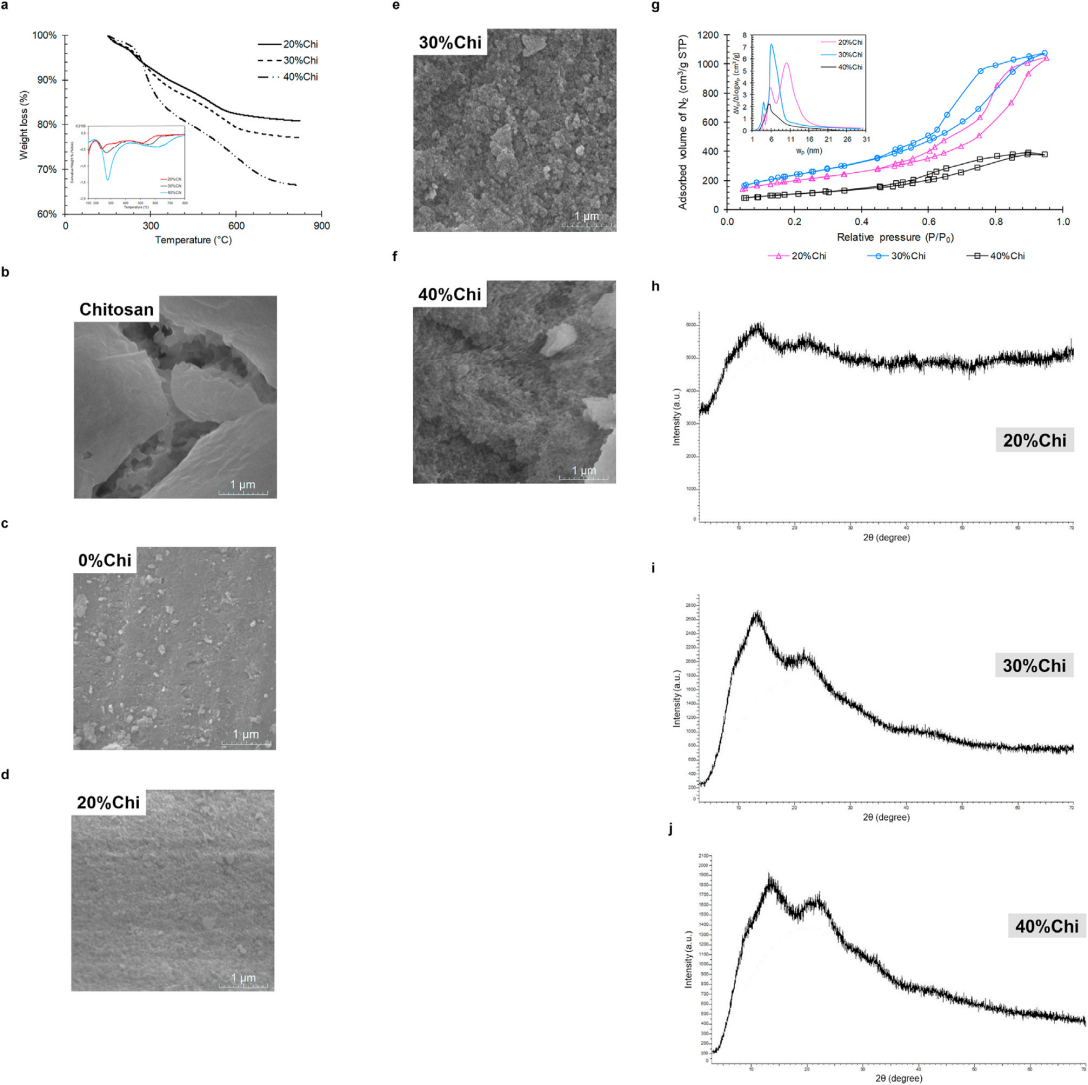
Physical characterization of the developed composites, being: (a) Thermogravimetric analysis, (b–f) Scanning electron microscopy increased by 120 kx, (g) Analysis of the surface area and the pore distribution, and (h–j) X-ray diffraction analysis.

Thermogravimetric analysis of the developed materials is shown in Figure 2 (a). It demonstrates that the residual material at 820 °C was 81%, 77% and 66% for 20%Chi, 30%Chi and 40%Chi composites, respectively. As can be observed, for the 20%Chi and 30%Chi composites, the residual mass percentages at 820 °C are between the silicate percentage values expected by stoichiometry and observed experimentally (Table 1). This obtained result confirms that not all TEOS has been converted to silicate. On the other hand, 40%Chi composite has a residual mass greater than the expected silicate content when observed in the mass balances. This suggests that with the increase in the chitosan content, the interactions between it and the composite are stronger, leading to greater thermal stability of the material [60].

Furthermore, thermal analysis shows that the increment in the amount of chitosan in the composite caused an increase in the temperatures of thermal degradation events. This is due to the silica condensation process and elimination of the hydroxyl group of chitosan [27, 55], giving better thermal stability to the composite. Diosa et al. [60] report that the reduction of chitosan in a silica network causes a less thermal degradation at temperatures between 520 and 650 °C.

Figure 2 (b-f) demonstrates the changes in the surface of each material regarding the introduction of chitosan, through the morphology of the materials. The images of the 20%Chi, 30%Chi and 40%Chi composites increased by 120 kx, demonstrate that there is a change in the surface of each material, with aspects of rough surfaces. It is observed in the images the rougher surface, characteristic of chitosan, for the 30%Chi and 40%Chi composites. On the other hand, for the 20%Chi composite, the surface is more compact and more similar to 0%Chi, due to the lower proportion of chitosan in this material. Budnyak et al. [27] and Ebisike et al. [55] reported similar observations on the development of organic adsorbents by the sol-gel system, including a hybrid chitosan/silica adsorbent by the sol-gel system.

In Figure 2 (g) it is possible to observe the adsorption isotherms from N_2_ to 77 K. From the BET theory, the surface areas of the developed composites were 718.15 m^2^/g, 886.31 m^2^/g and 374.94 m^2^/g for the 20%Chi, 30%Chi and 40%Chi composites, respectively. As for the pore distribution, the synthesized composites presented 9 nm, 7.5 nm and 6.2 nm for the 20%Chi, 30%Chi and 40%Chi composites, respectively, differing with the increment in chitosan and characterizing them as mesoporous (2–50 nm). Demonstrating that the increment in the amount of chitosan in silicate-based composites, especially above 30%, leads to an overlap of chitosan layers on the three-dimensional vacant silicate network. This causes a reduction in the composite surface area and a greater steric impediment from chitosan adsorption sites.

It is possible to observe that all samples have a type IV isotherm (Figure 2 (g)). Type IV isotherms are obtained when capillary condensation occurs, in which monolayer formation is observed, followed by adsorption of multilayers until inflection and saturation of the isotherm. Being typical of samples with pores in the range of mesopores to macropores, in which the formation of adsorption multilayers is possible, but the size of the porosity of the material is limited [61]. Furthermore, type IV isotherms feature a hysteresis loop (indicated in Figure 2 (g)), which appears when the adsorption and desorption curves do not coincide [62]. Present in mesoporous materials, with multilayer filling, which shows desorption at a pressure lower than the respective adsorption. Porous adsorbents, which have an unclear distribution of pore sizes and shapes have an H2 type hysteresis. This type of hysteresis was observed for both samples with different percentages of chitosan. With these results, it is possible to admit that the pores of the obtained materials are disordered and may be blocked, with the percolation phenomena [62].

Figure 2 (h-j) shows the X-ray diffraction analysis for the developed materials. From the diffractograms, the most prominent signs are observed at approximately 14° and 22° in the 2θ angle for the 20%Chi, 30%Chi and 40%Chi composites, indicating the amorphous state of the silica network [1, 55, 63].

### 3.2 pH effect in adsorption

**Figure 3:**
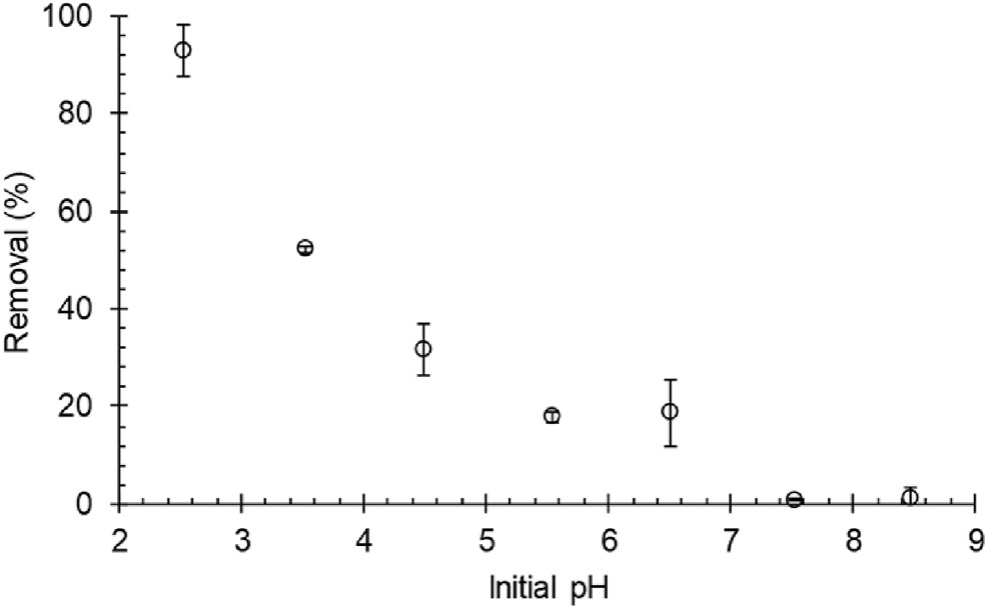
pH effect in the adsorption of the TY dye by the 30%Chi composite (25 °C, 100 rpm, 2 ​h, C_0_ = 100 mg/L and m/V = 0.05 g/L).

The pH effect is an important parameter for the adsorption technique when applied in the effluents treatment. Since the pH change can modify the disposition of the electronic charges of the adsorbate, influencing its ionization on the surface of the adsorbent. The pH effect in the adsorption of the TY dye is introduced in Figure 3.

According to Figure 3, the reduction in the pH of the solution caused an increment in the adsorption capacity of the adsorbent material. This is because, under pH conditions below pH_zcp_ (Figure 1 (e)) the surface of the adsorbent material is positively charged, mainly the chitosan amine groups present in the composite. This causes the electrostatic attraction of the solute, which has an anionic character, facilitating adsorption in acidic conditions. Similar results to this were observed by Huo et al. [22] and Georgin et al. [14], in which acidic conditions were favorable in the adsorptive processes. Under pH conditions above pH_zcp_ (Figure 1 (e)), in which the surface of the adsorbent is negatively charged, the adsorption capacity tends to zero, due to the repulsion of the (anionic) dye with the surface loads of chitosan.

### 3.3 Adsorption equilibrium

**Figure 4:**
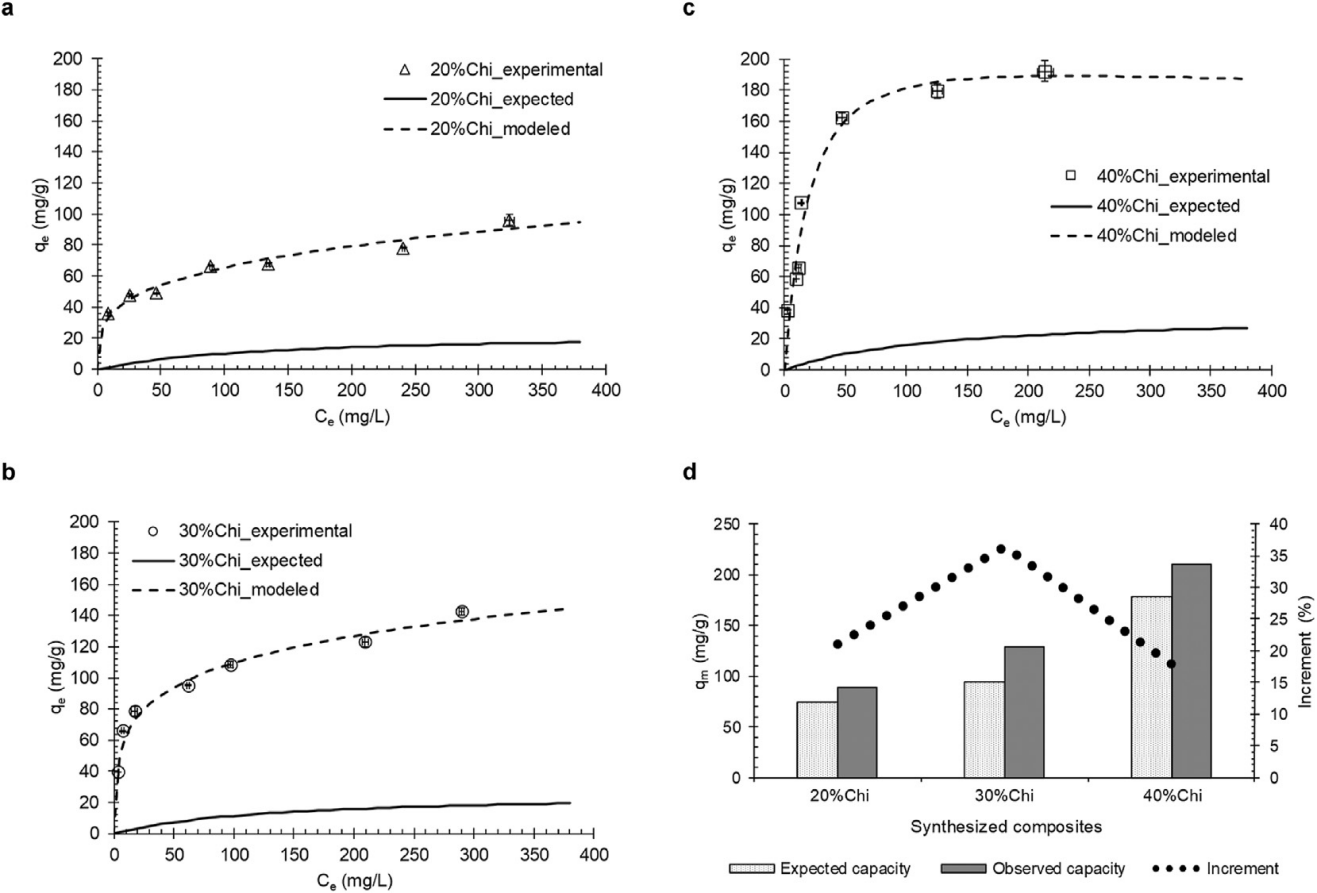
Adsorption isotherms of TY dye, being: (a) 20%Chi, (b) 30%Chi, (c) 40%Chi and (d) Expected and observed monolayer adsorption capacity for the developed composites (Table a.3). Solid lines represent the expected adsorption capacities (Eq. (1)). Dashed lines represent the Redlich-Peterson model fitting (Equation a.3) (25 °C, 100 rpm and pH = 2.5).

Adsorption isotherms provide information about the adsorbent, such as the maximum capacity and adsorption mechanism [64]. In addition, it relates the amount of adsorbent required to treat a given volume of solution up to an expected final concentration [42]. In Figure 4 (a, b and c) the adsorption isotherms of the TY dye by the developed composites are presented. It took approximately 24 h to reach experimental equilibrium. Isothermal data and modeling for the adsorption of chitosan and 0%Chi are presented in the supplementary material in the Table a.1 and a.2.

According to the experimental data in Figure 4 (a, b and c), is observed that the adsorption capacity of the composites was directly dependent on the amount of chitosan in the composite (40%Chi >30%Chi >20%Chi). This is due to the greater number of adsorption sites, related to the amine groups of chitosan. Piccin et al. [26] observed that the increase in the degree of deacetylation leads to an increment in the adsorption capacity of chitosan. This is due to a greater number of amine groups available as adsorption sites.

According to the classification of adsorption isotherms in liquid medium proposed by Giles et al. [65] the adsorption isotherms of the 20%Chi and 30%Chi composites are classified as L1 (Figure 4 (a) and (b)) and the isotherm of the 40%Chi composite (Figure 4 (c)) is classified as L2. The class of isotherms L means that the molecules are adsorbed on the surface of the adsorbent material, and as the empty spaces are being filled it becomes more difficult for the solute molecule to find an available location. Subclass 1 indicates that there is no monolayer formation and the adsorption sites are not completely occupied or that their availability decreased with increasing solute concentration [42, 65]. Type L1 isotherms were observed for the adsorption of acid red 97 dye by spores of the fungus *Beauveria bassiana* [14], azo dyes (TY and amaranth) by chitosan films [66] and in the removal of methylene blue dye per green coconut mesocarp [15]. Subclass 2, on the other hand, indicates that there is formation of monolayer in adsorption and complete saturation or unavailability of adsorption sites in certain concentrations [42]. Type L2 isotherms were observed for the adsorption of reactive black 5 dye by chitosan films [67], in the removal of the reactive blue 5G textile dyes by soybean hull treated with NaOH [68] and violet remazol 5R by chitosan [69]. The monolayer formation of the 40%Chi adsorbent composite (Figure 4 (c)), indicates that the increment in the amount of chitosan caused an increase in the adsorption capacity, due to the greater availability of active sites among the adsorbent with the TY dye. Table 2 presents the adjustment of the Langmuir, Freundlich and Redlich-Peterson models to the adsorption equilibrium data of the TY dye by the developed composites.

**Table 2:** Parameters of the Langmuir, Freundlich and Redlich-Peterson isotherm models for the adsorption of the TY dye by the developed composites (25 °C, 100 rpm and pH = 2.5).

Model	Parameters	Composite		
		20%Chi	30%Chi	40%Chi
Langmuir	K_L_ (mg/L)	0.039 ± 0.010	0.097 ± 0.020	0.055 ± 0.008
	q_m_ (mg/g)	89.3 ± 5.5	128.3 ± 5.5	209.9 ± 9.1
	R^2^	0.809	0.892	0.954
	Adjusted R^2^	0.793	0.883	0.950
Freundlich	K_F_ (mg/L)	18.28 ± 1.745	35.75 ± 2.270	38.08 ± 6.472
	n_F_	3.612 ± 0.249	4.173 ± 0.230	3.166 ± 0.376
	R^2^	0.955	0.973	0.887
	Adjusted R^2^	0.952	0.971	0.880
Redlich-Peterson	K_RP_ (mg/L)	373.420 ± 5.666	54.125 ± 3.345	9.952 ± 1.803
	a_RP_	20.220 ± 1.764	1.206 ± 0.912	0.029 ± 0.021
	β_RP_	0.725 ± 0.107	0.802 ± 0.028	1.089 ± 0.104
	R^2^	0.955	0.979	0.957
	Adjusted R^2^	0.946	0.975	0.951

According to the coefficients of determination R^2^ and the adjusted R^2^, it is observed that the Redlich-Peterson model presented the best fit (R^2^ > 0.95) to the experimental data of the TY dye for all developed composites. The Redlich-Peterson model is an empirical model with three parameters, and it is possible to represent the adsorption equilibrium over a wide concentration range, incorporating characteristics of the Langmuir and Freundlich models [41, 70]. This model suggests that there is greater surface variability of the adsorption sites, inferring that the sites are not homogeneous and that there is heterogeneity on the surface of the adsorbent [26]. Soon, the adsorption of the TY dye was governed by the formation of monolayer and interactions of multiple sites occurring concomitantly [71].

According to Table 2, the reduction of the K_RP_ and a_RP_ parameters indicate that the greater amount of chitosan in the composite caused a reduction in the adsorption affinity. This occurs because the reduction of chitosan reduces the steric impediment of the adsorption sites, leading to greater affinity. Similar results were found by Repo et al. [72] in the synthesis of chitosan/silica modified with EDTA (ethylenediamine tetra-acetic acid) in the adsorption of heavy metals (Co (II), Ni (II), Cd (II) and Pb (II)). Otherwise, the β_RP_ value close to 1 for the 40%Chi composite indicates the formation of monolayer (Table 2), simplifying the Redlich-Peterson model to that of Langmuir. β_RP_ values bottom than 1 are observed in the 20%Chi and 30%Chi composites (Table 2), indicating that the adsorption data did not favor the formation of the monolayer, corresponding to the Freundlich model. This demonstrates that the modulation of the amount of chitosan in the composite alters the arrangement of the adsorption sites.

In summary, it was observed that the 40%Chi composite has greater adsorption capacity, but that its sites are completely saturated. The question that remains, in this case, is: what is the ideal amount of chitosan to be added to the composite? For this purpose, the expected behavior of the isotherms is introduced by the solid lines in Figure 4 (d). This behavior was calculated from the adsorption isotherms of the constituent materials of the composite (chitosan and 0%Chi, presented in the supplementary material in the Table a.1 and a.2) and the respective formulation of the composites (Table 1), according to Eqs. (4) and (5). As noted, the synthesis method raised the adsorption capacity of all materials. This is explained by the fact that the synthesis method substantially increases the surface area of chitosan, exposing a greater number of adsorption sites and improving the morphology of the material. Similar results were observed by Budnyak et al. [27] in the development of a silica and chitosan composite for the adsorption of metal ions, in which they obtained improved adsorption capacities for the contaminants evaluated by means of the sol-gel system in comparison with the chitosan in natural form.

The maximum adsorption capacities of the monolayer (q_m_, estimated by the Langmuir model) obtained by the developed composites were compared with the expected values (Figure 4 (d)). It is noted that increments of maximum monolayer adsorption capacity were 18%, 36% and 21% for 20%Chi, 30%Chi and 40%Chi composites, respectively. This demonstrates that despite effectively increasing the adsorption capacity, with the increment of the amount of chitosan, part of the adsorption sites ends up being unavailable, making unfeasible the adsorption in them impossible. This is evident when comparing the surface areas of the materials (Figure 2 (g)), where the reduction is verified with the increase of the chitosan content.

The results achieved in this study present an adsorbent potential developed for the adsorption of emerging contaminants, as is the case of dyes, with relevant adsorption capacity, even when compared to other composite materials, as present in Table 3.

**Table 3:** Relation of the adsorption capacity of dyes using adsorbent composites reported in the literature.

Adsorbent	Adsorbate	q_m_ (mg/g)∗	Reference
30%Chi	Tartrazine yellow	128.3	This study
Activated carbon/chitosan/alginate	Remazol brilliant blue R	231.2	[2]
Cationic cellulose foam	Eosin Y	364.2	[6]
	Malachite green	193.8	
Magadiite/chitosan	Congo red	200.0	[7]
	Methylene blue	40.0	
Activated carbon derived from cassava sievate biomass by NaOH activation	Tartrazine yellow	106.9	[10]
	Methyl orange	161.8	
Binary oxidized cactus fruit peel	Brilliant green	166.7	[73]
Polyurethane/chitosan	Food red 17	55.0	[74]
Chitosan/pandan	Reactive black 5	169.5	[75]

∗ q_m_ is the maximum adsorption capacity, obtained according to the Langmuir isotherm model [39].

### 3.4 Adsorption kinetics

**Figure 5:**
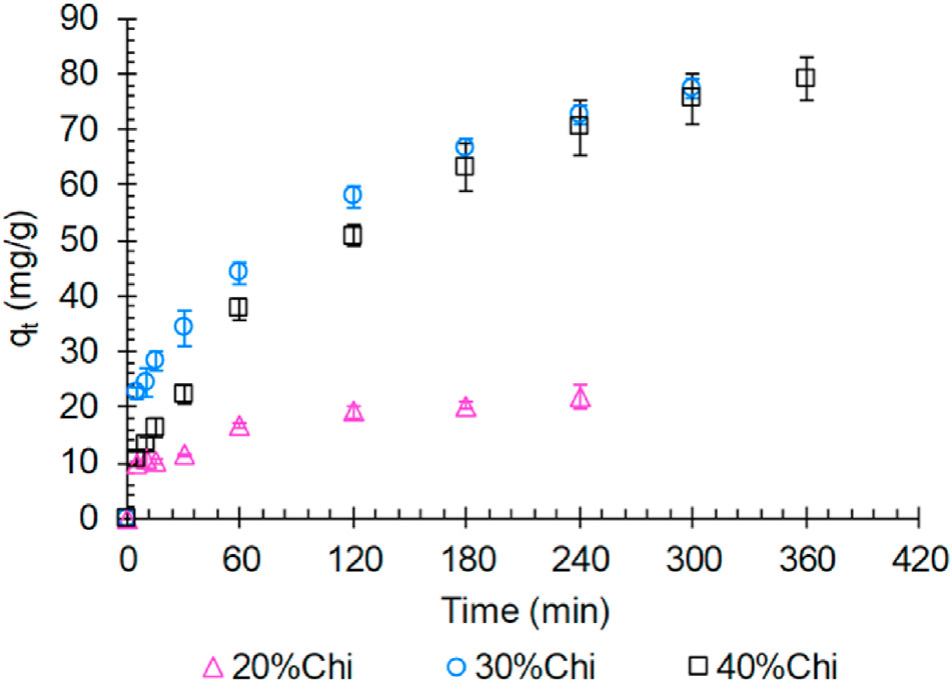
Adsorption kinetics of TY dye by the developed composites (25 °C, 100 rpm, pH = 2.5, C_initial_ = 100 mg/L and m/V = 0.3 g/L).

The TY dye adsorption kinetics by the developed composites is presented in Figure 5. According to Figure 5, the 20%Chi composite has an increase in the adsorption capacity (q_t_) during the first 60 min. After this period, there was a small variation in the adsorption capacity, demonstrating that this composite was practically saturated by the adsorbed dye, suggesting that the equilibrium has been reached. However, for the 30%Chi and 40%Chi composites, the adsorption capacity continued to increase during the experiments, indicating that the materials were not saturated by the adsorbed dye.

On the other hand, the delay in establishing the balance makes the composites 30%Chi and 40%Chi have an adsorption capacity approximately 4 times greater than 20%Chi. This increment in the adsorption capacity is related to the greater amount of chitosan present in these two materials (30%Chi and 40%Chi). However, the increment is not proportional, as observed in the adsorption isotherms, since the kinetics of 30%Chi and 40%Chi composites presented a similar behavior. This demonstrates that the 30%Chi composite has lower resistance to mass transfer, facilitating the access of the dye to the adsorption sites.

Table 4 presents the adjustment parameters of the Pseudo-first-order and Pseudo-second-order models to the kinetic data of the TY dye adsorption by the developed composites.

**Table 4:** Parameters of the Pseudo-first-order and Pseudo-second-order kinetics models for the adsorption of the TY dye by the developed composites (25 °C, 100 rpm, pH = 2.5, C_initial_ = 100 mg/L and m/V = 0.3 g/L).

Model	Parameters	Composite		
		20%Chi	30%Chi	40%Chi
Pseudo-first-order	k_1_∗1000 (min^−1^)	57.262 ± 11.536	24.879 ± 4.141	10.492 ± 1.058
	q_1_ (mg/g)	19.1 ± 1.1	69.2 ± 3.2	77.8 ± 2.6
	R^2^	0.826	0.896	0.976
	Adjusted R^2^	0.801	0.883	0.974
Pseudo-second-order	k_2_∗1000 (g/mg/min)	3.968 ± 0.265	0.409 ± 0.078	0.107 ± 0.015
	q_2_ (mg/g)	20.9 ± 1.1	78.8 ± 3.6	98.2 ± 4.0
	R^2^	0.900	0.942	0.985
	Adjusted R^2^	0.889	0.935	0.983

According to Table 4, the values of the coefficients of determination R^2^ and the adjusted R^2^ indicate that the TY dye adsorption kinetics is better described by the Pseudo-second-order kinetic model. The values of q_2_ are equivalent to the values of the experimental equilibrium capacity predicted by the model (data not presented). The Pseudo-second-order model suggests that the mass transfer during adsorption is controlled by external convection (film diffusion) and by diffusion inside the particle [45, 46]. Furthermore, through the kinetic constants (Table 3), it can be observed that the speed decreases with the greater amount of chitosan in the developed materials.

### 3.5 Adsorption thermodynamics

The effect of the temperature in the adsorption of the TY dye by the 30%Chi composite was evaluated in adsorption isotherm experiments, presented in the supplementary material in the Table a.4. The effects of temperature in the adsorption characteristics of a material are expressed in thermodynamic parameters of Gibbs free energy (ΔG^0^, Eq. (6)), entropy (ΔS^0^) and enthalpy (ΔH^0^). These parameters were calculated according to the Van't Hoff equation (Eq. (7)). This equation correlates the behavior of the equilibrium constant (K_D_), obtained by plotting ln(q_e_/C_e_) at C_e_ → 0 [76], as showed in Figure a.1 of the supplementary material. The thermodynamic parameters obtained from the Van't Hoff equation are shown in Table 5.

(6) $$\Delta G^{0}=-R\times T\times \text{ln}\left(K_{D}\right)$$

(7) $$ln\left(K_{D}\right)=-\frac{\Delta H^{0}}{R\times T}+\frac{\Delta S^{0}}{R}$$

where, R is the universal gases constant (8.314 J/Kmol), T is the temperature (K), K_D_ is the thermodynamic equilibrium constant (-), $\Delta {\text{H}}^{\text{0}}$ is the adsorption enthalpy and $\Delta {\text{S}}^{\text{0}}$ is the adsorption entropy.

**Table 5:** Thermodynamic parameters of the adsorption of the TY dye by the 30%Chi composite (100 rpm and pH = 2.5).

Temperature (K)	K_D_ (-)	$\Delta {\text{H}}^{\text{0}}$ (kJ/mol)	$\Delta {\text{S}}^{\text{0}}$ (J/mol K)	$\Delta {\text{G}}^{\text{0}}$ (kJ/mol)
288.15	9.951			-5.586
298.15	12.693	15.095	71.772	-6.304
308.15	13.946			-7.021

According to Table 5, the positive value of ΔH^0^ demonstrates that the adsorption is endothermic. The greatness of the adsorption enthalpy suggests that it occurs due to physical interactions [73], such as van der Waals forces, which are considered relatively weak interactions, but which are present in adsorptive processes [77], being favored at high temperatures. The increase in temperature can produce a swelling effect in the internal structure of chitosan, allowing the dye molecules to penetrate further into the structure of the adsorbent [78, 79]. Similar results were obtained by Schio et al. [74] in the synthesis of a foam composed of bio-based polyurethane/chitosan using ricinoleic acid for the adsorption of food red 17 dye. In addition, the negative ΔG^0^ values in the range of 15–35 °C and positive ΔS^0^ values demonstrate that the adsorption is spontaneous and disorder of the system increase during the adsorption. Moreover, entropy contributes more to the adsorption in this temperature range than the enthalpy.

### 3.6 Regeneration and reuse

**Figure 6:**
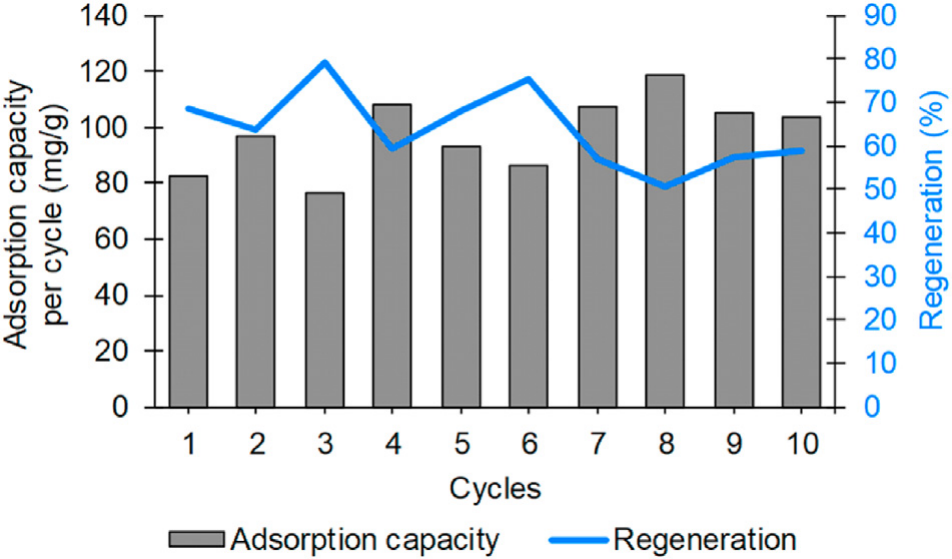
Reuse cycles of the 30%Chi composite using 0.05 mol/L NaOH eluent solution.

For possible applications in the treatment of effluents, the reuse of the adsorbents plays a significant role in the ecological and economic sense of the process. Adsorbent materials must be able to withstand several cycles of adsorption and desorption before their final destination. Figure 6 demonstrates the efficiency of the 30%Chi composite in removing the TY dye as a function of its reuse cycles.

According to Figure 6, it is possible to verify that the adsorption capacity varied between 76 and 118 mg/g, showing a tendency to increase with the number of cycles. Demonstrating that the use of 0.5 mol/L NaOH as an eluent in the desorption of the TY dye by the 30%Chi composite does not reduce the adsorption capacity of the material. In the presence of NaOH, the chitosan amine groups are deprotonated, leading to a breakdown of interactions with the dye. However, part of the dye may not be desorbed due to other interactions with the composite at alkaline pH. This was verified since the regeneration efficiency of the adsorbent varied between 79% and 50%.

Vieira et al. [78] evaluated the desorption and regeneration of chitosan coated glass beads for the adsorption of the TY dye using NaOH solutions (0.1, 0.25, 0.5 and 1.0 mol/L) as eluent. The authors observed that the increase in the concentration of the eluent favored desorption, reaching from 66% to 99%. The fixed bed regeneration reached up to four cycles, reaching rapid exhaustion in the last cycle.

Just like, Razmi et al. [75] using an adsorbent based on chitosan-modified pandan leaves, obtained 7 regeneration cycles in the removal of Reactive Black 5 dye using distilled water as an eluent. The authors observed that only in the first 5 cycles of adsorption the regeneration was greater than 50%, reducing to 34% and 28% in the following cycles.

## 4 Conclusion

The sol-gel system enabled the development of new adsorbents based on chitosan and silica, successfully combining the physical and chemical properties of these materials. The synthesis method allowed obtaining materials with a high surface area and exposing chitosan adsorption sites. The 30%Chi composite was the most efficient when subjected to adsorption of the TY dye, providing an increment in the maximum adsorption capacity in the monolayer of 36%. The life cycle analysis of the 30%Chi composite showed that the adsorption capacity was not reduced after 10 reuse cycles. Therefore, the new adsorbent composites can present excellent potential to be applied in the treatment of water and effluents. This study opens the possibility of investigating these composites in the adsorption of new emerging contaminants harmful to the ecosystem, and contributing with potential candidates for such application.

## Declarations

### Author contribution statement

Jonatan Rafael de Mello, Thaís Strieder Machado: Conceived and designed the experiments; Performed the experiments; Analyzed and interpreted the data; Wrote the paper.

Larissa Crestani, Ingridy Alessandretti: Conceived and designed the experiments; Performed the experiments.

Giovana Marchezi: Performed the experiments; Wrote the paper.

Flávia Melara: Performed the experiments; Analyzed and interpreted the data; Wrote the paper.

Marcelo Luis Mignoni: Analyzed and interpreted the data; Contributed reagents, materials, analysis tools or data; Wrote the paper.

Jeferson Steffanello Piccin: Conceived and designed the experiments; Analyzed and interpreted the data; Contributed reagents, materials, analysis tools or data; Wrote the paper.

### Funding statement

This work was supported by the Coordenação de Aperfeiçoamento de Pessoal de Nível Superior (CAPES - Finance Code 001), Conselho Nacional de Desenvolvimento Científico e Tecnológico (CNPQ – Proc. 405311/2016-8) and by the 10.13039/501100020832University of Passo Fundo.

### Data availability statement

Data included in article/supplementary material/referenced in article.

### Declaration of interests statement

The authors declare the following conflict of interests: This work is related to a patent filing application (Process number: BR 10 2021 003029 1).

### Additional information

No additional information is available for this paper.
