# Supplementary material for: Synthesis, characterization and application of new adsorbent composites based on sol-gel/chitosan for the removal of soluble substance in water
Source: Heliyon. 2022 May 17;8(5):e09444. doi: 10.1016/j.heliyon.2022.e09444 (PMC9127326; doi:10.1016/j.heliyon.2022.e09444)
Supplement: Supplementary material [file mmc1.docx]

**Supplementary material**

The Langmuir [1] isotherm model is described by Equation a.1:

$$q_{e}= \frac{K_{L}\times q_{m}\times C_{e}}{1+K_{L}\times C_{e}} (a.1)$$

where, q_m_ and q_e_ are the adsorption capacity at equilibrium and the maximum monolayer adsorption capacity (mg/g), K_L_ is the equilibrium constant of the Langmuir model (L/mg) and C_e_ is the equilibrium concentration in the liquid phase (mg/L).

The Freundlich [2] isotherm model is described by Equation a.2:

$$q_{e}= K_{F}\times C_{e}^{\frac{1}{n}} (a.2)$$

where, q_e_ is the maximum adsorption capacity at equilibrium (mg/g), K_F_ is the constant of the Freundlich model ((mg/g)(L/mg)^1/n^), 1/n is the heterogeneity factor and C_e_ is the concentration of the supernatant at equilibrium (mg/L).

The Redlich-Peterson [3] isotherm model is described by Equation a.3:

$$q_{e}=\frac{K_{RP}\times C_{e}}{(1+a_{RP}\times C_{e}^{\beta_{RP}})} (a.3)$$

where, q_e_ is the adsorption capacity (mg/g), K_RP_ and a_RP_ are the constants of the Rendlich-Peterson model (L/g or mg/L), C_e_ is the equilibrium concentration (mg/L) and β_RP_ is the exponent ranging from 0 to 1.

The Pseudo-first-order [4] kinetic model is described by Equation a.4:

$$q_{t}=q_{1}\left( 1-exp\left( -k_{1}t \right) \right) (a.4)$$

where, q_t_ is the adsorption capacity at time t (mg/g), q_1_ is the theoretical value of the adsorption capacity (mg/g), k_1_ is the Pseudo-first-order rate constant (min^-1^) and t is the time (min).

The Pseudo-second-order [5, 6] kinetic model is described by Equation a.5:

$$q_{t}=\frac{t}{\left( \frac{1}{k_{2}q_{2}^{2}} \right)+\left( \frac{t}{q_{2}} \right)} (a.5)$$

where, q_t_ is the adsorption capacity at time t (mg/g), t is the time (min), k_2_ is the Pseudo-second-order rate constant (g/mg/min) and q_2_ is the theoretical value of the adsorption capacity (mg/g).

The algorithms used in the modeling are available in https://www.dropbox.com/sh/elq2br3319ccbds/AAB2CTE2Hgb8bG_0Ue48tfLfa?dl=0.

**Table a.1.** Isothermal data for the adsorption of the TY dye by the base materials, chitosan and 0%Chi (25 °C, 100 rpm, m/V=0.05 g and pH=2.5).

| **Chitosan** | | **0%Chi** | |
| --- | --- | --- | --- |
| C_e_ (mg/L) | q_e_ (mg/g) | C_e_ (mg/L) | q_e_ (mg/g) |
| 116.604 | 301.643 | 389.683 | 12.517 |
| 128.467 | 290.319 | 390.551 | 11.454 |
| 119.714 | 205.707 | 295.069 | 11.312 |
| 124.850 | 197.701 | 294.201 | 12.497 |
| 57.9162 | 152.368 | 193.511 | 9.727 |
| 40.4353 | 120.996 | 193.559 | 9.655 |
| 39.249 | 122.048 | 137.523 | 8.928 |
| 18.605 | 78.899 | 88.191 | 6.226 |
| 17.968 | 78.968 | 88.885 | 5.452 |
| 11.554 | 64.980 | 64.176 | 4.346 |
| 16.193 | 60.795 | 39.003 | 4.334 |
| 7.918 | 37.814 | 38.627 | 4.760 |
| 6.086 | 39.652 | - | - |

**Table a.2.** Parameters of the Langmuir, Freundlich and Redlich-Peterson isotherm models for the adsorption of the TY dye by the base materials, chitosan and 0% Chi (25 °C, 100 rpm, m/V=0.05 g and pH=2.5).

| **Model** | **Parameters** | **Material** | |
| --- | --- | --- | --- |
|  |  | **Chitosan** | **0%Chi** |
| Langmuir | K_L_ | 0.011 | 0.007 |
|  | q_m_ | 433.262 | 16.747 |
|  | R^2^ | 0.895 | 0.945 |
|  | Adjusted R^2^ | 0.885 | 0.940 |
| Freundlich | K_F_ | 12.164 | 0.799 |
|  | 1/n_F_ | 1.594 | 2.153 |
|  | R^2^ | 0.901 | 0.934 |
|  | Adjusted R^2^ | 0.892 | 0.927 |
| Redlich-Peterson | K_RP_ | 263.542 | 0.123 |
|  | a_RP_ | 21.063 | 0.008 |
|  | β_RP_ | 0.377 | 0.981 |
|  | R^2^ | 0.901 | 0.945 |
|  | Adjusted R^2^ | 0.882 | 0.933 |

**Table a.3.** Monolayer adsorption capacities of the raw materials. The expected adsorption capacity of the composite was obtained by equation 1. The new adsorption capacity of chitosan was calculated based on the adsorption capacity of the developed composites.

|  | **Material** | | | | |
| --- | --- | --- | --- | --- | --- |
|  | **Chitosan** | **0%Chi** | **20%Chi** | **30%Chi** | **40%Chi** |
| Adsorption capacity in the monolayer (mg/g) | 433.262 | 16.746 | 89.33 | 128.297 | 209.948 |
| Expected adsorption capacity (mg/g) | - | - | 73.804 | 94.022 | 178.187 |
| New chitosan adsorption capacity (mg/g) | - | - | 652.109 | 691.521 | 541.666 |

**Table a.4.** Parameters of the Langmuir, Freundlich and Redlich-Peterson isotherm models for the adsorption of the TY dye by the 30%Chi composite at different temperatures (100 rpm, pH=2.5).

| **Model** | **Parameters** | **Temperature** | | |
| --- | --- | --- | --- | --- |
|  |  | **15°C** | **25°C** | **35°C** |
| Langmuir | K_L_ | 0,093 | 0.097 | 0.095 |
|  | q_m_ | 96.778 | 128.296 | 142.128 |
|  | R^2^ | 0.883 | 0.891 | 0.875 |
|  | Adjusted R^2^ | 0.873 | 0.882 | 0.864 |
| Freundlich | K_F_ | 31.671 | 35.752 | 43.603 |
|  | 1/n_F_ | 4.860 | 4.172 | 4.616 |
|  | R^2^ | 0.926 | 0.973 | 0.784 |
|  | Adjusted R^2^ | 0.920 | 0.970 | 0.766 |
| Redlich-Peterson | K_RP_ | 24.362 | 54.125 | 13.134 |
|  | a_RP_ | 0.546 | 1.206 | 0.087 |
|  | β_RP_ | 0.855 | 0.802 | 1.011 |
|  | R^2^ | 0.941 | 0.978 | 0.875 |
|  | Adjusted R^2^ | 0.930 | 0.975 | 0.852 |

**Fig. a.1.** Effect of the temperature in the equilibrium constant (KD) according to Van’t Hoff (Equation a.5) for the adsorption of the TY dye by the 30%Chi composite (100 rpm and pH=2.5).


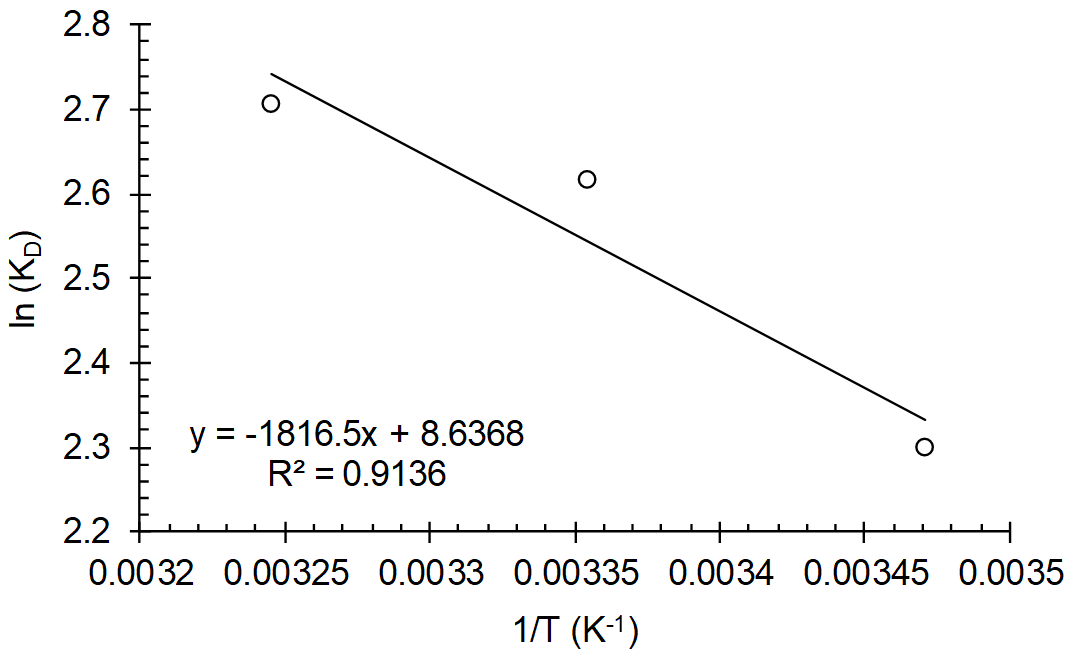


**References**

[1] Langmuir, I. (1918). The adsorption of gases on plane surfaces of glass, mica and platinum. *Journal American Chemical Society*, 40, 1361-1403.<https://doi.org/10.1021/ja02242a004>.

[2] Freundlich, H. M. F. (1906). Over the adsorption in solution. *The Journal of Physical Chemistry*, 57, 385-471.

[3] Redlich, O., & Peterson, D. L. (1959). A useful adsorption isotherm. *Journal of Physical Chemistry,* 63, 1024.<https://doi.org/10.1021/j150576a611>.

[4] Lagergren, S. (1898). Zur Theorie der Sogenannten adsorption geloster stoffe, Kungliga Svenska Vetenskapsakademiens. *Handlingar*, 24 (4), 1-39.

[5] Ho, Y. S., & Mckay, G. (2000). The kinetics of sorption of divalent metal ions onto sphagnum moss peat. *Water Research,* 34 (3), 735-742. https://doi.org/10.1016/S0043-1354(99)00232-8.

[6] Ho, Y. S, & Mckay, G. (1999). Pseudo-second order model for sorption processes. *Process Biochemistry,* 34, 451–465. https://doi.org/10.1016/S0032-9592(98)00112-5.
